# Using Re-Sampling Methods in Mortality Studies

**DOI:** 10.1371/journal.pone.0012340

**Published:** 2010-08-23

**Authors:** Igor Itskovich, Brad Roudebush

**Affiliations:** Underwriting Standards Department, Northwestern Mutual Life Insurance Company, Milwaukee, Wisconsin, United States of America; Tulane University, United States of America

## Abstract

Traditional methods of computing standardized mortality ratios (SMR) in mortality studies rely upon a number of conventional statistical propositions to estimate confidence intervals for obtained values. Those propositions include a common but arbitrary choice of the confidence level and the assumption that observed number of deaths in the test sample is a purely random quantity. The latter assumption may not be fully justified for a series of periodic “overlapping” studies. We propose a new approach to evaluating the SMR, along with its confidence interval, based on a simple re-sampling technique. The proposed method is most straightforward and requires neither the use of above assumptions nor any rigorous technique, employed by modern re-sampling theory, for selection of a sample set. Instead, we include all possible samples that correspond to the specified time window of the study in the re-sampling analysis. As a result, directly obtained confidence intervals for repeated overlapping studies may be tighter than those yielded by conventional methods. The proposed method is illustrated by evaluating mortality due to a hypothetical risk factor in a life insurance cohort. With this method used, the SMR values can be forecast more precisely than when using the traditional approach. As a result, the appropriate risk assessment would have smaller uncertainties.

## Introduction

A traditional and commonly used approach in mortality studies is based on the standardized mortality ratio (SMR) model, described in a number of texts [1]–[3]. The SMR is defined as the ratio of the observed to expected numbers of deaths and is often times expressed as follows:
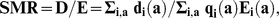
(1)where D and E are the total actual and expected numbers of deaths, summation is taken by selected equal time intervals i (usually, 1 year long durations) and relevant strata a (e.g., age groups), d_i_(a) = observed number of deaths, q_i_(a) = population death rate (conditional probability of death), and E_i_(a) = exposure (number of person-intervals) in i-th interval for stratum a. The values of other relevant classification variables, such as sex and smoking status, may be blended in eq. (1) or the SMR may be computed separately for each individual combination. The observed number of deaths in the numerator is taken for a sample with specific distinct characteristics (e.g., a certain disease), whose relative mortality is to be assessed in the study.

The above approach provides a simple way to evaluate mortality ratios for wide range of study conditions, with a direct method of estimating standard errors based on the binomial distribution. For large enough numbers of deaths in each individual group (d_i_(a)> = 5, q_i_(a)E_i_(a)> = 5) and total population size much larger than the size of the study sample, a simple approximation, based on the normal distribution of the observed numbers of deaths [4], may be used:
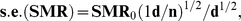
(2)where SMR_0_ is given by (1), d = Σ_i, a_ d_i_(a) is total observed number of deaths and n is the size of the study sample. The resultant confidence interval for SMR may be approximated as follows:
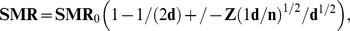
(3)where Z is the normal distribution score for selected confidence level (Z = 1.96 for commonly used 95% confidence level).

An alternative, so-called Byar's [3], approximation is a simple but amazingly precise approximation to exact results based on the Poisson distribution:
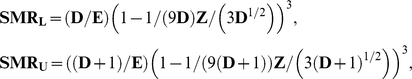
(4)where SMR_L_, SMR_U_ are the lower and upper limits of the confidence interval.

## Methods

### Issues with Conventional Approach

Although simple and straightforward, these methods of estimating confidence intervals have a number of weaknesses. The first obvious issue is the necessity to select a particular confidence level. Even though the 95% value is a standard selection in most situations, there is no solid justification for using it; any choice is, strictly speaking, an arbitrary one. The second issue, that is the center point of the present article, is the most general assumptions upon which these traditional estimation methods are based. It is always assumed that the actual number of deaths in a population for which the final SMR is evaluated is a purely random quantity. This means that the true number can have any value according to the corresponding distribution with the mean equal to the observed number of deaths in the study sample. While this assumption is quite reasonable for prediction attempts that stretch indefinitely into the future, in many real situations it would be too conservative resulting in unjustifiably wide confidence intervals. Indeed, often times it is more meaningful to attempt forecasting relevant SMR values only over a finite number of years of additional follow-up. One possible reason is the need for most accurate and credible SMR estimates over a relatively narrow time horizon. Plausible examples are: repeated studies of the effect of pollutants in a community, recurring studies of occupational mortality, or an ongoing study of a particular risk factor found in individual life insurance underwriting. Each of these studies would use as its expected mortality basis a (potentially stratified) set of mortality rates representative of the population the study subjects were drawn from.

For instance, consider a typical mortality study conducted for entrants to a study within the past 10 years with a maximum 10 year observation period, and assume that the observed number of deaths is 400. If the objective of the study is to forecast the SMR 3 years into the future when a new 10 year observation period will be used, it would be too much of a stretch to assume that the confidence interval for the observed number of deaths is given by 400(1+/−1.96/(400)^1/2^) = 400+/−39 (see Eq. 3, assuming 1/(2d)≈0, (1−d/n)^1/2^≈1). Indeed, since the next 10 year study will include all deaths from last 7 years of entrants with a maximum 7 year follow-up that are included in the current one, let's say, 250 deaths, only about 150 or so “new” deaths may have to be treated as a random variable, and a more reasonable final estimate might look like 250+150(1+/−1.96/(150)^1/2^) = 400+/−24 deaths. Furthermore, the assumption that all 150 “new” deaths are a purely random quantity is an exaggeration. Since the majority of those deaths (perhaps, 120 or so) will come from the same 7 years of entrants followed for additional 3 years, their number will not be completely independent of the 250 deaths included in the current study. Only a few additional deaths (say, about 30) coming from 3 years of new entrants followed for up to 3 years total will have to be treated as a completely independent random variable.

### Proposed New Method

Obviously, developing a rigorous approach based on sound statistical methods to incorporate these ideas may not be a feasible task. Luckily, there is no need to embark upon such a difficult journey. The center piece of the present paper is a new proposed approach that is very straightforward, independent of any statistical assumptions, and may therefore be justifiably called “evidence-based”. The approach we are proposing here is based on a generalization of re-sampling methods that have gained some recognition in the last decades. Extensive literature now in existence [4]–[6] describes specific methods (e.g., bootstrap and jackknife), developed by a number of scholars, and offers sophisticated arguments, based on sound statistical principles, in support of those methods. The main difference among various re-sampling approaches lies in the specific way that the set of test samples is created and in the number of those samples that is deemed appropriate to provide desired credibility and precision of the resultant estimates. Our method is free of any ambiguity associated with both those complications due to the presence of a natural time scale provided by the study itself. In the example discussed earlier, the relevant scale is the length of the period for consecutive studies (e.g., 3 years).

Once that main time interval has been specified, the selection of the test samples becomes most straightforward. Specifically, in the proposed re-sampling method, every possible sample falling within the said time interval is used. Consecutive samples are obtained simply by censoring the original sample back one day (or other smallest time increment available with the data) at a time. Censoring in this context means setting the end of the study on a specified day and excluding all deaths that occurred on later days from the analysis. Simultaneously, the start date of the study is also adjusted on a daily basis in such a way that a specified constant length of the follow-up (observation period) is always maintained. Therefore, for granular enough data, with all relevant dates (entering/leaving the study or death) for each subject known to the nearest day, the number of test samples is equal to the total number of days in the study's main time interval.

## Results

### Example: Risk Factor Study

In order to illustrate the proposed method, let us set up a mortality study with the purpose of evaluating a risk factor in a cohort of life insurance applicants. It matters not what the risk factor is, merely that it may infer additional mortality compared to the baseline mortality rates derived from the entire cohort. The question at hand will be if this risk factor's extra mortality varies by age.

The experience here is taken from data available to the authors encompassing fully underwritten insurance policies issued between 1996–2008 from which the base death rates (quantities q_i_(a) in Eq. 1) were derived. The observation period for the study will be set at 10 years, and we will present results for a 3-year main time interval (forecasting horizon). The risk factor in this mortality study is comprised of selected impairments that are routinely underwritten and for which extra premium is typically assessed. All the analyses have been performed using SAS programming language, version 8.2.

The study has been run with two consecutive data samples: one with an observation period of 1996–2005, another one with an observation period of 1999–2008. The characteristics of the two samples are presented in Table 1; there have been no significant changes in the distribution by sex and age over the chosen 3-year time interval. Table 2 below demonstrates the results obtained by running the 10 year mortality studies in a traditional way, according to Byar's approximation (Eqs. 4). In order to make the results as “credible” as reasonably possible, the study population was limited to (middle-age) adult nonsmokers, ages 30–69. Eight 5-year wide age groups a (30–34, 35–39, …, 65–69) were used for the base death rates and SMR computations, with 1-year long time intervals i to account for effects of varying durations in eq. 1. The death rates were always computed for the corresponding 10 year study sample, and no attempts have been made to smooth them out in any way. All the results presented below are for the exposures computed at the beginning of 1 year time intervals; using mid-interval exposures instead yields practically identical results. In the interests of protecting the company's proprietary data, all SMR values have been “normalized” by representing them as ratios to the SMR value for the entire study sample (both sexes, ages 30–69).

**Table 1:** Study samples for two 10-year observation periods, three years apart.

		1999–2008		1996–2005	
Sex	Age	Cases	Deaths	Cases	Deaths
All	30–69	134,979	679	145,085	702
All	30–49	103,162	348	111,934	333
All	50–69	31,817	331	33,151	369
Male	30–49	79,464	290	85,282	284
Male	50–69	23,798	261	24,728	300
Female	30–49	23,698	58	26,652	49
Female	50–69	8,019	70	8,423	69

**Table 2:** Traditional approach for two 10-year observation periods, three years apart.

		1999–2008		1996–2005	
Sex	Age	SMR	95% C.I.	SMR	95% C.I.
All	30–69	1.00	0.93–1.08	1.00	0.92–1.08
All	30–49	1.12	1.00–1.24	1.05	0.95–1.21
All	50–69	0.90	0.80–1.00	0.96	0.83–1.05
Male	30–49	1.07	0.95–1.20	1.03	0.92–1.19
Male	50–69	0.87	0.77–0.98	0.95	0.81–1.05
Female	30–49	1.47	1.11–1.90	1.25	0.88–1.71
Female	50–69	1.02	0.80–1.29	0.98	0.76–1.31

SMR = Standardized Mortality Ratio, C.I. = SMR Confidence Interval.

All values normalized by the SMR for the entire study sample (both sexes, ages 30–69).

With this hypothetical study, the main issue under investigation is whether the risk assessment of this factor should be made dependent on age. The most general observation of Table 2 results immediately raises the following question: are the SMR values for ages 30–49 and 50–69 significantly different? Based on unisex analysis, and for the 1999–2008 observation period, we might conclude that they are, since the corresponding 95% level confidence intervals (1.00–1.24 for ages 30–49 and 0.80–1.00 for ages 50–69) do not overlap. However, looking at the 1996–2005 observation period, we find that the intervals do overlap, and the conclusion drawn from that earlier study should be: no statistically significant difference between the two age bands. Furthermore, when stratified by sex, the confidence intervals for the two age groups in question overlap noticeably for both study re-runs. Therefore, in a real-life situation, the conclusion would likely be that no stratification by age was justified. The 1999–2008 study might draw attention to the first disputable evidence for emerging possible age dependence of the risk factor. It is likely, though, that only in case of clearly non-overlapping confidence intervals repeated for several subsequent re-runs would stratification by age be seriously considered.

The situation is substantially different with the proposed new approach used for the estimation of the confidence intervals in question. Table 3 below presents the results obtained by using re-sampling method described earlier for the 1999–2008 observation period with daily re-sampling, that is, setting the beginning and ending censor date forward one day at a time from the 1996–2005 observation period until covering the 1999–2008 period. There are in effect then 1,096 resulting ten year observation periods using this technique. The average SMR is taken from these individual observation period SMRs and the endpoints of the confidence intervals correspond to the minimum and maximum values of the SMR distribution.

**Table 3:** Proposed 10-year observation period, with three years of daily re-sampling.

		Observation period progressing daily from 1996–2005 to 1999–2008	
Sex	Age	SMR	Full C.I.
All	30–69	1.00	0.97–1.03
All	30–49	1.11	1.05–1.16
All	50–69	0.91	0.86–0.99
Male	30–49	1.07	1.02–1.12
Male	50–69	0.89	0.82–0.99
Female	30–49	1.38	1.19–1.61
Female	50–69	1.00	0.94–1.09

SMR = Mean of SMR distribution, Full C.I. = Min−Max of SMR distribution.

All values normalized by the SMR for the entire study sample (both sexes, ages 30–69).

## Discussion

In accordance with earlier discussion, each confidence interval turns out to be much narrower than in the traditional approach. As a result, none of them overlap, even when stratified by sex, and the only possible conclusion could be the positive need for age stratification.

A natural question arises: how robust is the proposed method, e.g., what is the minimum sample size and/or number of deaths that can assure the credibility of the corresponding estimates? For this evidence-based approach and in the absence of any other model parameters except the main time interval (3 years) and the length of the follow-up (10 years), we suggest a simple practical criterion. Namely, as long as the actual consecutive study design ensures large enough overlapping (7 years out of 10 in the current example) that the re-sampling confidence intervals are significantly tighter than the ones resulting from the traditional approach, the proposed method may be regarded as meaningful and preferred. Of course, depending on the specific statistic under investigation, the relative tightness of the confidence intervals may or may not matter for the final study results. For example, let's now consider a 5-yr study with the same main time interval of 3 years. As presented in Table 4, the re-sampling confidence intervals for the two age groups widen sufficiently to overlap for both sexes. And even though they are still significantly tighter than those obtained with the traditional approach, the proposed method does not provide any added value – the conclusion would still be: no stratification by age justified at this point.

**Table 4:** Proposed 5-year observation period, with three years of daily re-sampling.

		Observation period progressing daily from 2001–2005 to 2004–2008	
Sex	Age	SMR	Full C.I.
All	30–69	1.00	0.90–1.10
All	30–49	1.12	0.85–1.25
All	50–69	0.90	0.76–1.02
Male	30–49	1.15	0.89–1.35
Male	50–69	0.86	0.72–1.05
Female	30–49	0.94	0.51–1.66
Female	50–69	1.04	0.75–1.42

SMR = Mean of SMR distribution, Full C.I. = Min−Max of SMR distribution.

All values normalized by the SMR for the entire study sample (both sexes, ages 30–69).

Similarly, if a 10-yr study is re-run with one additional year of daily re-sampling (4-yr main time interval), questions about the need for stratification by age arise. As Table 5 shows, even though for males the confidence intervals still do not overlap, they now do for females. Therefore, the simplest, most conservative, conclusion may be that for a 10-yr study the longest forecasting horizon ensuring the robustness of the proposed method and reliability of its predictions is 3 years.

**Table 5:** Proposed 10-year observation period, with four years of daily re-sampling.

		Observation period progressing daily from 1996–2005 to 2000–2009	
Sex	Age	SMR	Full C.I.
All	30–69	1.00	0.97–1.03
All	30–49	1.11	1.05–1.16
All	50–69	0.91	0.86–0.99
Male	30–49	1.07	1.03–1.12
Male	50–69	0.88	0.79–0.99
Female	30–49	1.41	1.19–1.61
Female	50–69	1.06	0.95–1.26

SMR = Mean of SMR distribution, Full C.I. = Min−Max of SMR distribution.

All values normalized by the SMR for the entire study sample (both sexes, ages 30–69).

At the same time, a more aggressive modification of the proposed method may be justified. It has to do with the fact that so far we have evaluated the confidence intervals over the entire forecasting horizon (3 or 4 years), which resulted in their lower and upper limits being constant. However, the statistic under investigation – SMR – can clearly be time-dependent. Indeed, a quick look at Table 2 data suggests, for example, that for females, especially those ages 30–49, this dependence may, in fact, be quite significant. Therefore, a natural refinement of the proposed method could consist of dividing the original main time interval into a number of smaller intervals and performing described daily re-sampling over each one of them separately. This way, the corresponding number of SMR values with their associated confidence intervals will result. If plotted as a function of a time variable describing each individual re-sampling interval by a single point (e.g., the end of its observation period), the time-dependent confidence bands will be generated, similar to those produced with the Principal Response Curves (PRC) method [7]. Consider, for example, using 1-yr individual re-sampling intervals. As shown in Table 6, the corresponding confidence intervals do not overlap for either one of the sexes, and the resulting “confidence bands” do not overlap anywhere. An obvious question now arises: in what case can this modified approach work and how should the specific individual re-sampling intervals be adequately chosen? Clearly, in order for this refined method to be justified, there should be some systemic, rather than just random, variations in SMR value for those individual intervals. With our example, such differences could readily be caused by changes in the underwriting standards that result in varying sample selections over the years.

**Table 6:** Four proposed 10-year observation periods, with one year of daily re-sampling each.

		Observation period from 1999–2008 to 2000–2009		Observation period from 1998–2007 to 1999–2008		Observation period from 1997–2006 to 1998–2007		Observation period from 1996–2005 to 1997–2006	
Sex	Age	SMR	Full C.I.	SMR	Full C.I.	SMR	Full C.I.	SMR	Full C.I.
All	30–69	1.00	0.98–1.02	1.00	0.98–1.02	1.00	0.98–1.02	1.00	0.98–1.02
All	30–49	1.13	1.10–1.18	1.13	1.11–1.16	1.11	1.09–1.12	1.08	1.04–1.10
All	50–69	0.90	0.87–0.92	0.89	0.86–0.91	0.91	0.89–0.92	0.94	0.89–0.98
Male	30–49	1.07	1.04–1.13	1.09	1.05–1.12	1.06	1.04–1.08	1.05	1.02–1.09
Male	50–69	0.83	0.80–0.85	0.86	0.83–0.88	0.89	0.87–0.90	0.93	0.87–0.98
Female	30–49	1.49	1.37–1.60	1.42	1.33–1.56	1.46	1.32–1.62	1.27	1.18–1.42
Female	50–69	1.22	1.15–1.27	1.02	0.96–1.09	1.03	0.98–1.07	0.96	0.93–0.99

SMR = Mean of SMR distribution, Full C.I. = Min−Max of SMR distribution.

All values normalized by the SMR for the entire study sample (both sexes, ages 30–69).

An example of computing the confidence bands for PRC by performing a standard non-parametric bootstrap at each time point (weekly data were used) is given in [8]. It is instructive to compare the results obtained by using our proposed method and the standard bootstrap. Table 7 presents the results of applying bootstrap re-sampling to the two 10-yr study samples discussed earlier. Even though only 100 re-samples were used, the confidence intervals are much wider than those for the proposed method and significantly overlap for both sexes (with more commonly used 1000 re-samples, the confidence intervals would be even wider). Bootstrap re-samples were drawn using PROC SURVEYSELECT in SAS, with Unrestricted Random Sampling (URS) method, i.e., with replacement.

**Table 7:** Standard Bootstrap with 100 re-samples for the two 10-year observation periods.

		1999–2008		1996–2005	
Sex	Age	SMR	Full C.I.	SMR	Full C.I.
All	30–69	1.00	0.91–1.09	1.00	0.89–1.11
All	30–49	1.13	0.95–1.28	1.05	0.90–1.20
All	50–69	0.90	0.76–1.07	0.96	0.86–1.10
Male	30–49	1.11	0.95–1.29	1.07	0.92–1.23
Male	50–69	0.87	0.72–1.05	0.98	0.85–1.15
Female	30–49	1.22	0.76–1.61	0.97	0.70–1.43
Female	50–69	1.00	0.72–1.33	0.87	0.61–1.17

SMR = Mean of SMR distribution, Full C.I. = Min−Max of SMR distribution.

All values normalized by the SMR for the entire study sample (both sexes, ages 30–69).

Another issue, that standard re-sampling methods concern themselves with, is that of a possible bias in the obtained estimate of the statistic in question, i.e. the difference between its empirical (sample-based) value and the “true” (population-based) value. There are ways to estimate that bias for various re-sampling methods that are described in the literature [4]–[6]. The advantage of the simple approach proposed in the present paper is the absence of any “true” value for the statistic under investigation (SMR), except that obtained with the daily re-sampling over the appropriate time interval. In other words, the described full set of daily re-samples represents the entire “population” over the specified time horizon. The only unknown part of this “population” is the one that will appear in subsequent studies as a result of newly underwritten future cases. Of course, there is no rigorous way to make any credible inferences regarding the future samples updated with those cases.

To further demonstrate the benefits of the proposed method, let us also apply the traditional approach of Eq. 4 to the entire sample with 13-yr long observation period (1996–2008). As demonstrated in Table 8, with the numbers of deaths almost twice those for each one of the two 10-yr studies (see Table 2), the confidence intervals become much tighter. As a result, for males they do not overlap anymore although they touch. But for females the confidence intervals for the two age groups still overlap, and so even three additional years of observation do not provide clear indication of the need for stratification by age.

**Table 8:** Traditional approach for the entire 13-year observation period.

Sex	Age	Cases	Deaths	SMR	95% C.I.
All	30–69	170,397	1208	1.00	0.94–1.06
All	30–49	128,663	604	1.11	1.02–1.20
All	50–69	41,734	604	0.91	0.84–0.99
Male	30–49	98,680	498	1.08	0.99–1.18
Male	50–69	31,132	477	0.90	0.82–0.99
Female	30–49	29,983	106	1.27	1.04–1.54
Female	50–69	10,602	127	0.95	0.79–1.13

SMR = Standardized Mortality Ratio, C.I. = SMR Confidence Interval.

All values normalized by the SMR for the entire study sample (both sexes, ages 30–69).

The other obvious result here is that with tighter confidence intervals, the specific SMR values can be forecast more precisely than when using the traditional approach. As a result, with the proposed method used, the appropriate risk assessment would have smaller uncertainties.

### Concluding Remarks

One final comment: so far, we have not mentioned a powerful method, based on the Cox proportional hazards model [9], [10], that lately has been increasingly used in epidemiological mortality studies. That model has been gaining popularity in bio-medical research over the traditional SMR approach because of its exceptional versatility. The main advantages of the Cox model are: its suitability for multivariate analysis, no need for any specific assumptions regarding survival probability distribution in the base population, and the ability to handle time-dependent variables. However, the estimated confidence intervals for computed hazard ratios are generally at least as wide as those for the SMR model and depend on a number of statistical assumptions, including the aforementioned need to select a specific confidence level. The reason for the confidence intervals being especially wide is the presence of intrinsic uncertainties associated with the maximum likelihood method which is an integral part of any practical quantitative implementation of the Cox model [10]. As a result, it suffers from the same weaknesses discussed earlier as the SMR approach, which may also substantially reduce its predictive power in a number of important situations.

As an illustration, in Table 9 we present the results from multivariate Cox regression obtained for the most recent re-run of the same 10-year study. The analysis was conducted using PROC PHREG in SAS and, wherever appropriate, included multivariate sex/age adjustments. It is clear that even for that re-run, which, with the traditional SMR approach used, gave the first indication of possible need for age stratification, the Cox regression would not discover statistically significant difference between the two age groups. Similar results would be obtained with the earlier study re-run. Therefore, even though the proposed approach could easily be used with the Cox regression (by re-sampling successive re-runs of PROC PHREG), that would not provide any additional benefit in the model's predictive power.

**Table 9:** Cox model for 10-year observation period.

		1999–2008	
Sex	Age	HR	95% C.I.
All	30–69	1.00	0.91–1.09
All	30–49	1.09	0.97–1.23
All	50–69	0.90	0.79–1.02
Male	30–49	1.08	0.95–1.23
Male	50–69	0.86	0.75–1.00
Female	30–49	1.15	0.87–1.52
Female	50–69	1.01	0.77–1.33

HR = Hazard Ratio, C.I. = HR Confidence Interval.

All values normalized by the HR for the entire study sample (both sexes, ages 30–69).

In summary, the new approach to estimating confidence intervals for the SMR values in mortality studies by employing re-sampling methods, proposed in the present article, may provide important advantages over the traditional approach based on the binomial/Poisson distribution for the observed numbers of deaths.
